# Exploring inclusiveness towards immigrants as related to basic values: A network approach

**DOI:** 10.1371/journal.pone.0260624

**Published:** 2021-12-02

**Authors:** Hadi Sam Nariman, Lan Anh Nguyen Luu, Márton Hadarics

**Affiliations:** 1 Doctoral School of Psychology, ELTE Eötvös Loránd University, Budapest, Hungary; 2 Institute of Psychology, ELTE Eötvös Loránd University, Budapest, Hungary; 3 Faculty of Education and Psychology, ELTE Eötvös Loránd University, Budapest, Hungary; 4 Department of Social Psychology, ELTE Eötvös Loránd University, Budapest, Hungary; Rzeszow University of Technology: Politechnika Rzeszowska im Ignacego Lukasiewicza, POLAND

## Abstract

Using the 9^th^ round of European Social Survey (ESS), we explored the relationship between Europeans’ basic values and their attitudes towards immigrants. Employing a latent class analysis (LCA), we classified the respondents based on three items capturing the extent to which participants would support allowing three groups of immigrants to enter and live in their countries: immigrants of same ethnic groups, immigrants of different ethnic groups, and immigrants from poorer countries outside Europe. Four *classes* of Europeans with mutually exclusive response patterns with respect to their inclusive attitudes towards immigrants were found. The classes were named Inclusive (highly inclusive), Some (selective), Few (highly selective), and Exclusive (highly exclusive). Next, using a network technique, a partial correlation network of 10 basic human values was estimated for each class of participants. The four networks were compared to each other based on three network properties namely: *global connectivity*, *community detection*, and *assortativity coefficient*. The global connectivity (the overall level of interconnections) between the 10 basic values was found to be mostly invariant across the four networks. However, results of the community detection analysis revealed a more complex value structure among the most inclusive class of Europeans. Further, according to the assortativity analysis, as expected, for the most inclusive Europeans, values with similar motivational backgrounds were found to be interconnected most strongly to one another. We further discussed the theoretical and practical implications of our findings.

## Introduction

Immigration is still among the most controversial topics in the Western political sphere. On the one hand, right-wing parties and populist leaders center their rhetoric around anti-immigration sentiments (see e.g., [1,2]), and on the other hand, anti-immigration attitudes are a significant motive behind supporting and voting for right-wing parties (e.g., [3–6]). In recent years, the number of immigration-related research has significantly increased, perhaps partly due to the above-mentioned reasons.

The interplay between a variety of contextual and individual level factors has been argued to explain anti-immigrant attitudes and opposition to immigration. Poor economic condition of the host society [7], anti-immigration media portrayals [8–10], immigration-related restrictive policies [11,12], regional origin of migrants [13], and immigrants’ actual population size [14] are among the contextual factors on mobilizing anti-immigrant attitudes. Among the individual level predictors of anti-immigrant attitudes are perceived economic threat and competition [15,16], perceived cultural threat [17,18], perceived threat vis-à-vis safety and security [19–21], nativist perception of national identity [22,23], ideological orientations such as right-wing authoritarianism and social dominance orientation [24], stereotypical judgements [25,26], low degrees of personality traits such as openness to experience and agreeableness[27], perceived size of immigrants’ population [28], low degree of interpersonal trust [29,30], and low degree of trust in national institutions [31] (for an overview see also [32]). Furthermore, tapping into a more positive aspect of immigration, it is noteworthy that groups of immigrants who are economically and culturally perceived as less threatening and more beneficial (e.g., immigrants from western societies vs. asylum seekers) receive more positive evaluations from the members of the host society [33,34].

In addition to the factors outlined above, it is also well-documented that the individual differences on basic personal values are strongly related to the endorsement of positive or negative attitudes and behaviors towards minority group members in general and immigrants in particular (see below). In the present study, using nationally representative samples, we draw on Schwartz’s [35] well-established and cross-culturally validated [36] value map, as related to Europeans’ inclusive attitudes towards immigrants. By inclusive attitudes, we mean the extent to which one would support allowing three groups of immigrants to enter their country—immigrants of same ethnic groups, immigrants of different ethnic groups, and immigrants from poorer countries outside Europe.

Applying a network method, the objective of this study is to explore and compare the dynamic structure of the basic personal values held by different *classes* of Europeans with qualitatively different inclusive attitudes towards immigrants. Our study, therefore, comprises two analytical phases. First, a person-centered method (latent class analysis) is applied for the classification of the respondents. Second, partial correlation networks are estimated to further investigate the dynamic structure of the basic personal values for each distinct class found in the first phase of the analyses. Below we discuss the relationship between personal values and attitude towards immigrants as well as both of the analytical approaches in more detail.

### Basic personal values and anti-immigrant attitudes

**Fig 1:**
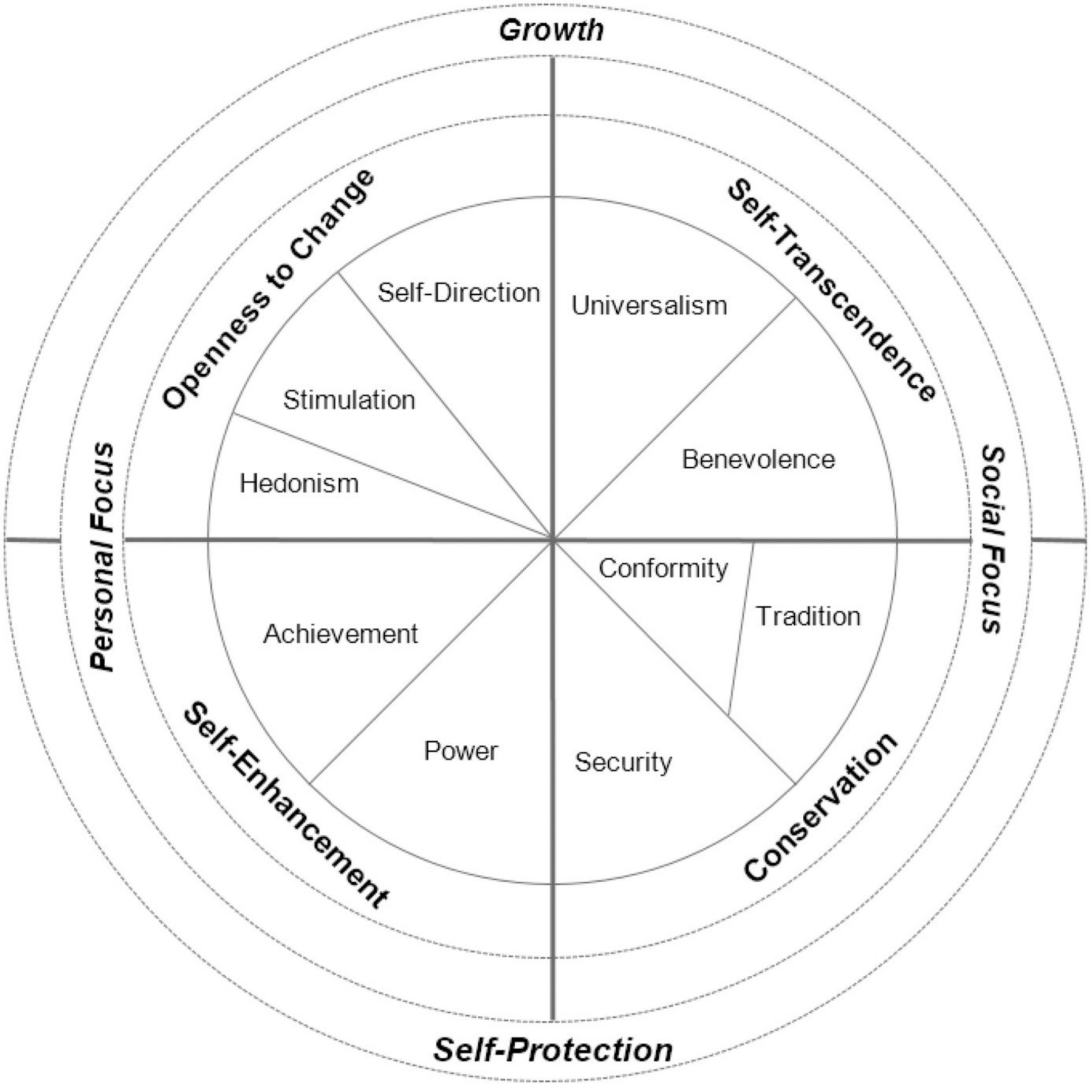
Schwartz value circle depicting the relations between 10 values and several value groupings [35,40].

Values are defined as “*desirable transsituational goals*, *varying in importance*, *that serve as guiding principles in the life of a person or other social entity*” [37, p. 21]. They are abstract and superordinate standards that determine individuals’ worldviews, attitudes, and behaviors in a vast array of more specific situations and contexts [35,38]. Schwartz [35], proposes a comprehensive value system into a circular dynamic of 10 basic personal values, representing the adjacent values capturing more similar motivational states and the values distant from each other to be motivationally more dissimilar or even antagonistic (see Fig 1, adapted from [39]).

The circumplex structure of the Schwartz’s values allow them to be categorized into 4 higher-order value types; Openness to change (Hedonism, Stimulation, Self-direction), Conservation (Security, Conformity, Tradition), Self-Transcendence (Universalism, Benevolence), and Self-Enhancement (Achievement, Power) (See supplementary material for the conceptual definitions of the 10 basic values). A pair of opposing value types (openness to change vs. conservation and self-enhancement vs. self-transcendence); in turn form two higher-order dimensions of values. Openness to change values focus on independence and seeking new experience and ideas in life, opposing conservation values that refer to the motivational goals of protecting social norms and traditions. On the other dimension, self-transcendence values tap into the well-being of other people, opposing self-enhancement values that focus on one’s own personal interests and welfare [35]. The 10 basic values can also be grouped into two other alternative higher-order value dimensions. Either as Social Focus (self-transcendence, conservation) versus Personal Focus (openness to change and self-enhancement), or Self-Protection (self-enhancement, conservation) versus Growth (openness to change, self-transcendence) [40]. In what follows, the emphasis of this article is on the latter distinction, between self-protection and growth categories, as two motivationally opposing higher-order value types.

Furthermore, empirical investigations have well-documented that self-protection value types positively and growth value types negatively relate to anti-immigrant attitudes (see e.g., [34,41–45]).

### Person-centered approach

Person-centered methods treat the individuals and not the variables as the units of analysis. This allows researchers to raise questions which cannot be answered by the more traditional variable-centered methods. By having at least two indicators (i.e., observed variables), it is possible to investigate whether there exist unobserved latent classes or subgroups of individuals whose response patterns are unique and qualitatively distinct from each other [46]. Classifying respondents into a set of mutually exclusive classes, for example, makes it possible to investigate what factors precede class membership, what the consequences are, and how class membership relates to demographic as well as contextual correlates. Person-centered methods can be especially useful and highly informative as long as the data represent the corresponding population. By a representative sample one would be more confident that the nature of the classes as well as their association with other constructs correspond to those of the population of interest (For an overview on person-centered approach in social psychological research see [47]). A person-centered method, therefore, is suitable for the first phase of our analyses. That whether there exist distinct classes of Europeans with meaningfully different response patterns regarding their inclusive attitudes towards the three groups of immigrants.

Person-centered methods have been recently applied in intergroup relation research. For example, Adelman and Verkuyten [48], found four distinct profiles with combinations of prejudice against Muslims and the level of participants’ tolerance on different Muslim practices (e.g., celebrating Islamic holidays in public). Through a variable-centered method, for instance, it was possible to regress the acceptance of Muslim practices on prejudice against Muslims. By a person-centered method, apart from finding profiles of people who were both prejudiced and against Muslim practices, they also found profiles of respondents who objected against some Muslim practices while not being particularly prejudiced. Another example is a study by Sibley et al. [49], where they found five distinct constellations of right-wing authoritarianism (RWA) and social dominance orientation (SDO). Among the five, three classes were found to be low, mild, or moderate on both SDO and RWA. In line with the assumption made by Altemeyer [50], they also found two other classes of respondents: authoritarian followers (Low SDO and High RWA) and authoritarian leaders (High SDO and low RWA). While the positive correlation between SDO and RWA is well-documented (e.g., [51]), by applying a person-centered method, was it possible to find profiles of respondents being low in SDO but high in RWA or the other way around.

With regards to the present study, instead of applying a person-centered method, one possibility was to divide the participants arbitrarily. For example, based on median splits, it is possible to create classes of participants with all the possible combinations of high, moderate, and low levels of the indicator variables. However, since we used nationally representative samples, as mentioned above, one could be more confident that the response patterns found by the person-centered technique would be consistent with those of the corresponding population (European population in the case of the current paper). Further, research indicates that splitting the data by median would increase the risk of power loss and the emergence of spurious effects (see [52]).

### Network analysis

Network methods are a suitable technique for conceptualizing a number of psychological variables as a complex system in which every single variable is in direct associations with all the other variables. Unlike a latent model, for example, that assumes the observed variables to measure the latent variable equivalently, networks allow picturing the dynamic of all the existing unique and direct pairs of interconnections [53]. More traditional correlation-based research is often parsimonious and uses a limited number of variables for analysis. Modeling a relatively larger number of variables as a network, enables researchers to have a more comprehensive picture of all the interrelationships. Network methods have been recently employed in multiple domains of psychological research, investigating, for instance, personality traits [54], mental disorders [55], and stereotype structure [56]. More specifically, attitudes towards immigrants have been also investigated through network models (see e.g., [25,57]).

Nodes and edges are the most primary constituent elements of a network. Typically, in psychological models, nodes are a set of observed variables and edges the direct and unique statistical associations (i.e., partial correlations) between every possible pair of edges [58].

Connectivity is another basic network property that refers to the extent to which the nodes within a network are interconnected. In psychological networks, connectivity refers to the level of causal interdependencies between a set of nodes. The stronger the interconnection between two nodes, the more likely it is that changes to one node will also result in changes to the other. This is because as two cognitive components are strongly related to each other they need to maintain consistency, so changes to one cognitive component (smoking causes cancer or not?) should lead to changes to the other (should I smoke or not?) and vice versa [59–61]. Thus, in strongly connected networks, the nodes yield stronger level of causal effect on each other, while in weakly connected networks they would behave more accidentally and less dependent upon the impact of one another (for a detailed overview on the relationship between network connectivity and causality in network science and its integration with psychological networks see [62]). Furthermore, previous research shows that as one holds a strong attitude, different kinds of one’s evaluations on the corresponding attitude object, are more strongly interconnected or causally interdependent. In other words, the connectivity between an attitude’s underlying components (i.e., cognitive, affective, and behavioral) predicts attitude strength towards the attitude object (see the Causal Attitude Network (CAN) model, [63]). For example, the connectivity between a set of anti-Roma evaluations was found to be stronger for those who held stronger attitude towards the Roma people [64]. Moreover, a group of nodes being strongly interconnected, manifests their belonging to a similar state [62]. Global connectivity, for instance, as one measurement of network connectivity, refers to the absolute sum of all the (partial) correlation coefficients within a network.

As mentioned above, Schwartz [35] proposes the 10 basic values in a circular structure suggesting a dynamic of relations between the values. Network analysis, hence, would be an efficient method to representing and further investigating this dynamic. An extensive body of research has tested the relationship between the 10 basic values and intergroup attitudes (*cf*. *supra*). In the current study, we go beyond by exploring the relationships between the 10 basic values, estimated as partial correlation networks, for different classes of Europeans with unique inclusive strategies towards immigrants.

It is worth noting that person-centered methods are typically applied in exploratory research. Network methods have been recently used in exploratory papers as well (see for example [65–67]). In spite of the exploratory nature of our study, however, we hold two main hypotheses.

Concerning the person-centered analysis, although there are no theory-driven explanations regarding the exact number and nature of the classes, we follow previous research on expecting a number of classes aligned along a spectrum from the most inclusive to the most exclusive. For example, Morselli and Passini [68], found six profiles of Europeans (using the 6^th^ round of ESS data) on inclusiveness towards immigrants and protest against institutional authority—ranging from inclusive protestors to exclusive protestors. Our first assumption, therefore, is that there is a continuum of distinct typologies of individuals’ inclusiveness towards immigrants from the most inclusive to the most exclusive.

Moreover, a recent study found that compared to conservatives, in the network of liberals’ moral values, the interconnections within each set of more egalitarian (i.e., individualizing) and less egalitarian (i.e., binding) moral values was stronger than the interactions between them [69] (for an overview on binding and individualizing moral values see [70]). That is to say, in the liberal moral system, the values within each of the individualizing or binding categories, were found to be more interconnected while the interconnections between them was comparatively weaker. Past research shows that individualizing moral values negatively and binding moral values positively associate with intergroup prejudice (see eg., [71–73]). Also, compared to conservatives, liberals tend to express lesser degree of generalized prejudice [74] and evaluate different outgroups more positively (e.g., [75–77]. One, therefore, may argue that liberals evaluate the outgroup more positively, employing a set of strongly interconnected individualizing moral values, and their evaluation is more independent from the causal effect of other binding moral values. It is also worth pointing out that, generally, individualizing moral values are related to growth value types and binding moral values are related to self-protection value types (see for example [78]). Thus, our second hypothesis is that in the most inclusive group’s value network, the interconnections within each set of more egalitarian (i.e., growth) and less egalitarian (i.e., self-protection) basic values are the strongest and the interconnections between them are the weakest.

## Data and methods

Our study is based on nationally representative interview-based survey data using the 9^th^ round of European Social Survey (ESS), edition 1.2, collected in 2018. Verbal informed consents were obtained from all participants prior to the interviews being conducted. A total of 19 European countries participated in the data collection process: Austria, Belgium, Bulgaria, Cyprus, Czechia, Estonia, Finland, France, Germany, Hungary, Ireland, Italy, Netherlands, Norway, Poland, Serbia, Slovenia, Switzerland, and United Kingdom. Since we were interested in the adult population, out of the initial number of participants (N = 36015), we removed 1070 participants because they were under eighteen years old. Overall, 34945 participants (M_age_ = 51.6, SD = 18.1; 52.9% women) were included in the analyses. Table 1 reports the sample sizes and the descriptive statistics for the participants’ age and gender per country.

**Table 1:** Sample sizes and descriptive statistics for age and gender by country.

Country	*N*	% Females	*M*_age_ (*SD*)
Austria	2416	54.1	52.2 (17.5)
Belgium	1689	50.8	49.1 (18.5)
Bulgaria	1949	54.1	55.7 (16.9)
Cyprus	760	52.9	54.8 (18.3)
Czechia	2356	55.9	49.3 (17.3)
Estonia	1843	56.3	51.7 (18.6)
Finland	1674	51.2	52.1 (18.2)
France	1928	54.5	53.2 (18.4)
Germany	2258	49	50.8 (18.3)
Hungary	1603	57	51.7 (18.0)
Ireland	2140	52.6	52.6 (17.4)
Italy	2 617	52.8	51.9 (18.9)
Netherlands	1569	50.4	50.2 (17.8)
Norway	1308	44.6	48.4 (17.5)
Poland	1416	52.4	48.8 (18.1)
Serbia	1962	51.5	54.0 (17.5)
Slovenia	1260	53.8	50.4 (18.1)
Switzerland	1440	49.7	48.7 (18.1)
United Kingdom	2135	54.7	52.9 (18.1)

*Note*. *M* and *SD* indicate mean and standard deviation respectively.

### Measures

The indicator variables used in the classification procedure (latent class analysis) are three items measuring respondents’ support for allowing three groups of immigrants to enter their countries: (1) “To what extent do you think [country] should allow people of the same race or ethnic group as most [country]’s people to come and live here?” (2) “How about people of a different race or ethnic group from most [country] people?” (3) “How about people from the poorer countries outside Europe?”. All the three items were measured on a four-point scale (1 = Allow many to come and live here; 2 = Allow some; 3 = Allow a few; 4 = Allow none). In order to avoid possible misclassification of the respondents, we also controlled for the effects of a number of covariates namely political ideology, interest in politics, anti-immigrant evaluations, personal values, and participants’ demographic characteristics (gender, age, and education).

Political ideology was measured on an 11-point scale (“In politics people sometimes talk of “left” and “right”. Where would you place yourself on this scale, where 0 means the left and 10 means the right?”).

Interest in polities was measured on a four-point scale (“How interested would you say you are in politics?”) from *very interested* (1) to *not at all interested* (4).

Anti-immigrant attitudes were measured by economic threat perception (“Would you say it is generally bad or good for [country]’s economy that people come to live here from other countries?”), symbolic threat perception (“Would you say that [country]’s cultural life is generally undermined or enriched by people coming to live here from other countries?”), and a more general anti-immigrant attitude (“Is [country] made a worse or a better place to live by people coming to live here from other countries?”). All the three items were 11-point scales form *Bad for the economy*/*Cultural life undermined*/*Worse place to live* (0) to *Good for the economy*/*Cultural life enriched*/*Better place to live* (10) respectively.

Personal values were measured by the 21‐item Portrait Values Questionnaire (PVQ‐21, [79]). Each item portrays a person for whom a certain motivational value type is important and asks the respondent about the extent to which they find themselves similar, from *Very much like me* (1) to *Not like me at all* (6). For instance, “[She]He thinks it is important that every person in the world should be treated equally. [She]He believes everyone should have equal opportunities in life.”, is one of the items tapping into universalism. Each of the 10 basic values was measured by two items—universalism by three. The 10 basic values, therefore, were first used as the covariates for classifying the participants, and later they were estimated as partial correlation networks to picture the dynamic of each group’s personal values.

### Latent class analysis

Using Mplus software, version 8 [80], we applied a latent class analysis, a model-based person-centered method, to classify the respondents on the basis of their inclusiveness towards immigrants—using the three items mentioned above. Since apart from detecting the latent classes, we were also interested in the effect of our covariates on the participants’ class membership, we used a 3-step latent class method suggested by [81]. It is suitable for both detecting the latent classes, based on class membership probabilities, as well as controlling for the effect of covariates in determining the classes. Employing this method, first, the latent class model is estimated only based on the indicator variables. In the second step the most likely class membership variable is generated. And in the third step the class membership variable is regressed on the covariates, while also any potential misclassification occurred in the second step is fixed (see also [82]). In addition to the covariates mentioned above, we also created 18 country dummies (Slovenia as the reference group) to account for the country effects.

Further, since the three-step method is sensitive to missing values (on the covariates) and to avoid listwise deletion of the observations, the dataset was imputed. The proportion of the missing values was under 5 per cent for all the items used in the study ranging from 0% to 4.2% (except for political ideology with 13.8% missing values). Following [83], we applied a two-level multiple imputation technique, taking into account the participants’ being nested into their countries—assuming the data to be missing at random.

In order to find the best model solution, we first built a 2-class model, and increased the number of classes up to a 6-class model and compared the 5 models with each other. We decided on the best model solution based on statistical criteria, parsimony, and interpretability [84]. Four statistical criteria were used: Bayesian Information Criterion (BIC), Akaike’s Information Criterion (AIC), entropy, and the Vuong–Lo–Mendell–Rubin likelihood ratio (VLMR) test. The entropy value ranges from 0 to 1, the closer it is to 1, to a higher extent it indicates reliability of the classification, and separability between the classes [85]. The VLMR test compares the k-class model with the k-1-class model and provides a *p*-value to test whether the k-class model fits the data significantly better [86].

Moreover, to refrain from the impact of sampling biases on the classification of the respondents, the data was weighted. Because the sample sizes for each country are similar but the population sizes are different, the data was weighted by population to avoid the overrepresentation of smaller countries. We also weighted the data by design, correcting for the fact that the likelihood of the respondents to be represented in the data varies by country (see [87]).

### Network analysis

The 10 basic values were estimated as partial correlation networks for each class of the respondents we found in the previous stage. After having checked for the accuracy of the edge weights, the networks were explored and compared based on global connectivity, community detection, and assortativity coefficient.

#### Network estimation

To estimate the networks, for each class, the correlations between the 10 basic values were computed and inverted into partial correlations to obtain all the existing unique and direct statistical associations. We used *ggmModSelect* function in R from “qgraph” package [88]. The function first runs the *graphical least absolute shrinkage and selection operator* (glasso) algorithm for estimating 100 different network models with 100 different tuning parameters. Next, glasso fits the networks on *unregularized Gaussian graphical models* (GGM) and choses the best model based on BIC. Lastly, glasso adds and removes edges until it finds the model with the best BIC value.

#### Global connectivity

The global connectivity of the networks was computed and compared to each other using the R package “NetworkComparisonTest” (NCT) [89]). Permutations tests, with 1000 iterations, were run to check whether the overall level of interconnections is invariant across the networks.

#### Community detection

A community (or cluster) is a group of nodes that are densely connected to each other and are more weakly connected to other nodes in the network. It is, therefore, possible that different sets of highly connected nodes form a number of sub-networks within the network. To find the communities in the networks we used *Walktrap* algorithm from the “igraph” R package [90].

#### Assortativity coefficient

As mentioned above we assumed that in the network of the most inclusive class, the interactions between growth and self-protection value types would be the weakest and the interactions within each category would be the strongest. In order to test our assumption, first, using R package “assortnet” [91], we computed assortativity coefficient that quantifies the extent to which groups of nodes (growth vs. self-protection) within a network tend to be interacting within rather than between each other [92]. The assortativity coefficient ranges from -1 to 1 and values closer to 1 reflect the stronger tendency of nodes to interact within-group rather than between-group. Next, using r package “boot” [93] we compared the networks by calculating 95% confidence intervals obtained from 1000 bootstrap resamples.

#### Network stability

Prior to the main analyses, we performed a bootstrapping technique, checking for the accuracy of the edge weights. Using R package “bootnet” [58], bootstrapped confidence intervals around the edge weights were obtained from 1000 draws. A network is considered having accurate and therefore interpretable edge weights if the confidence intervals indicate low variabilities (see [58]).

## Results

### Latent class analysis

Table 2 summarizes the model fit comparisons between the 5 models. Compared to the 2-class and 3-class models, the 4-class model solution was found to be better fitting the data in terms of the statistical criteria. Only the entropy of the 3-class model (.92) was similar to that of the 4-class model (.91), while other statistical criteria showed significant improvement of the 4-class model fit. Further, in spite of the slight decrease of the AIC and BIC values in the 5-class and 6-class models, VLMR test showed that increasing the number of classes higher than 4 does not significantly improve the model fit. Moreover, compared to the 4-class model, the entropy value in the 5-class model solution (.79) and 6-class model solutions (.74) dropped sharply. Hence, we chose the 4-class model as the best in fitting the data, parsimoniousness, and interpretability.

**Table 2:** Model fit comparisons from 2-class to 6-class model solutions.

Number of classes	AIC	BIC	entropy	VLMR (*p*-value)
2	223449	223610	.89	< .001
3	200344	200589	.92	< .001
**4**	**190610**	**190940**	**.91**	**< .001**
5	190519	190933	.79	.06
6	190429	190928	.74	.81

*Note*. AIC and BIC decrease as the model fit improves.

As can been seen in Table 3, in the 4-class model, the probabilities of the respondents’ answers showed that in each class the majority of the respondents fall into one of the four categories of the three indicator variables. As mentioned above the four categories were: allow many, allow some, allow a few, and allow none. In each class the respondents were found to be consistent on their inclusiveness towards the three groups of immigrants. Therefore, supporting our first assumption, one class was found to be the most inclusive regarding all the three immigrant groups, one found to be the most exclusive, and the other two were found to be selective. We named the 4 classes as *Inclusive* (N = 4607), *Some* (N = 14399), *Few* (N = 10987), and *Exclusive* (N = 4952) (see the supplementals for the descriptive statistics and the correlations between the variables for each class).

**Table 3:** The probabilities of the participants’ responses on their inclusive towards immigrants by class membership.

Indicatior Variables	Class			
	Inclusive	Some	Few	Exclusive
imsmetn				
Allow many	**98%**	16%	7%	4%
Allow some	2%	**81%**	35%	14%
Allow a few		0%	2%	**56%**	25%
Allow none	0%	0%	1%	**57%**
imdfetn				
Allow many	**94%**	1%	0%	0%
Allow some	5%	**93%**	4%	0%
Allow a few	0%	6%	**89%**	3%
Allow none	0%	1%	7%	**97%**
impcntr				
Allow many	**82%**	3%	1%	0%
Allow some	15%	**80%**	10%	3%
Allow a few	2%	15%	**75%**	8%
Allow none	1%	2%	14%	**89%**

*Note*. imsmetn = Allow many/few immigrants of same race/ethnic group as majority; imdfetn = Allow many/few immigrants of different race/ethnic group from majority; impcntr = Allow many/few immigrants from poorer countries outside Europe.

Table 4 reports the results of the multinomial logistic regressions for class membership predicted by the covariates (with the Inclusive class as the reference category). Exclusive class members were found to be significantly more interested in politics, while political interest did not predict Some and Few class memberships significantly. Moreover, political conservatism and the three items tapping into threat perception significantly predicted Exclusive, Few, and Some class memberships. Moreover, concerning the self-protection value types, the importance of security and tradition negatively predicted Exclusive, Few, and Some class memberships. Conformity negatively predicted only the Exclusive class membership, and achievement negatively predicted only the Some class membership. Moreover, power did not associate with the three class memberships compared to the Inclusive class. Regarding the growth value types, benevolence and universalism positively predicted Exclusive, Few, and Some class memberships. Hedonism, stimulation, and self-direction were not found to be predictive of the participants class memberships. Regarding the demographic variables, participants’ gender (women as the reference category) significantly and negatively associated with the Few and Some class memberships and age positively predicted the membership of the Exclusive, Few, and Some classes. Further, the level of participants’ education negatively and significantly predicted the Exclusive and Few class memberships (see also S6 Table in the supplementals for the demographic characteristics of each class).

**Table 4:** Results of multinomial logistic regressions for predicting class membership by the covariates (Inclusive class as the reference category).

Variables	Exclusive	Few	Some
Political Interest	.24***	.08	-.02
Political Ideology	.28***	.25***	.15***
Imbgeco	-.68***	-.41***	-.21***
Imueclt	-.49***	-.38***	-.22***
Imwbcnt	-.50***	-.35***	-.17***
Gender	-.17	-.24***	-.18***
Age	.03***	.03***	.02***
Education	-.08***	-.03***	-.006
Security	-.32***	-.29***	-.22***
Conformity	-.15***	-.05	.001
Tradition	-.11*	-.13***	-.10**
Benevolence	.26***	.26***	.13*
Universalism	.93***	.67***	.40***
Self-direction	.09	.06	.04
Stimulation	-.02	-.02	-.006
Hedonism	-.03	-.03	.03
Achievement	-.08	-.07	-.08*
Power	-.07	-.06	-.03

*Note*.

*** = *p* < .001

** = *p* < .01

* = *p* < .05. imbgeco = immigration is good or bad for economy; imueclt = whether immigration undermines or enriches culture; imwbcnt = immigration makes the country better or worse place to live.

Before proceeding to the next stage of the analyses we also performed a series of independent sample t-tests to compare the four classes based on political ideology, interest in politics, perceived threat towards immigrants, and the 10 basic values. The results showed that the four classes (Inclusive, Some, Few, and Exclusive) ranged from the most liberal and least interested in politics to the most conservative and most interested in politics respectively and all the differences were statistically significant. The four classes were also significantly different from each other regarding the three items measuring threat perception, with the Inclusive class to be the lowest on threat perception (i.e., Inclusive < Some < Few < Exclusive). Similarly, the extent to which the respondents found growth value types important, ranged from the highest for the Inclusive class to the lowest for the Exclusive class (Some and Few in between, respectively). This pattern was exactly the opposite concerning the self-protection value types. Only, regarding achievement, no significant difference was found in none of the comparisons. Also, the difference between the Few and Exclusive groups on tradition was not found to be significant (see the supplementals for the t-test results).

### Network analysis

**Fig 2:**
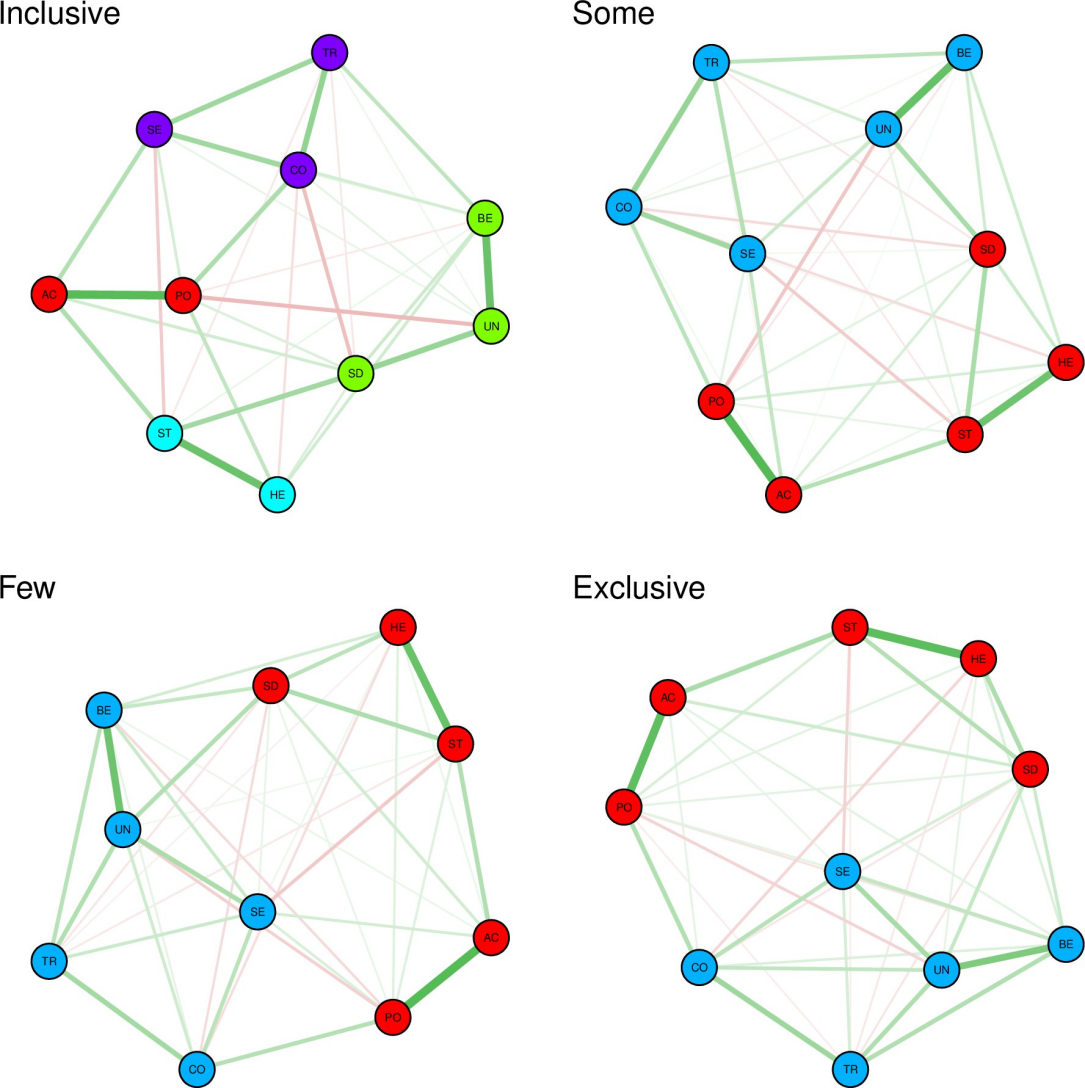
Partial correlation networks estimated for the 4 classes found in the LCA. Red lines depict negative partial correlations, and the green lines represent positive partial correlations. Node with the same color belong to the same community. The thickness of the lines represents the magnitude of the correlation coefficients. SD = Self-Direction; ST = Stimulation; HE = Hedonism; AC = Achievement; PO = Power; SE = Security; CO = Conformity; TR = Tradition; BE = Benevolence; UN = Universalism.

Four networks were estimated based on the respondents’ 10 basic values (see Fig 2). For each network, out of 45 possible edges, we checked for the number of non-zero edges (Inclusive = 33, Some = 39, Few = 38, and Exclusive = 38).

#### Global connectivity

The global connectivity scores were found to be more or less similar across the 4 networks (Inclusive = 5.16; Some = 5.14; Few = 5.31; Exclusive = 5.34). The results of the permutation tests showed that, out of 6 possible comparisons only the Few-Some difference was found to be significant (Inclusive vs. Some: diff = .02, *p* = .92; Inclusive vs. Few: diff = .15, *p* = .19; Inclusive vs. Exclusive: diff = .18, *p* = .06; Some vs. Few: diff = .16, *p* = .02; Some vs. Exclusive: diff = .20, *p* = .05; Few vs. Exclusive: diff = .03, *p* = .72).

#### Community detection

As Fig 2 represents, the community detection analysis showed that in Some, Few, and Exclusive networks, personal focus values vs. social focus values formed 2 large separate communities. While the nodes in Inclusive network tended to form 4 communities, highly corresponding to the four higher order value types: openness to change, self-transcendence, self-enhancement, and conservation. The only slight difference was that self-direction clustered with self-transcendence values: universalism and benevolence.

#### Assortativity coefficient

The differences between the 4 networks on the assortativity coefficient were all above chance (Inclusive: r = .81, 95% CI [.79, .84]; Some: r = .66, 95% CI [.64, .67]; Few: r = .51, 95% CI [.48, .53]; Exclusive: r = .39, 95% CI [.35, .43]). Our second hypothesis was therefore supported, that compared to other networks, in Inclusive network, the interconnections within the growth value types and within the self-protection value types was the strongest, and the interconnections between the two categories was the weakest.

#### Network stability

The stability analyses showed that all the networks were stable and therefore interpretable regarding their edge weights. That is to say, the confidence intervals around the edge weights showed small variabilities, meaning that the edge weight accuracy was attained across all the 4 networks (figures visualizing the confidence intervals around the edge weights can be found in the supplementals).

## Discussion

Using Latent class analysis, we analyzed the 9^th^ round of ESS data, and found 4 mutually exclusive classes of Europeans with meaningfully distinct inclusive attitudes towards the three groups of immigrants: from the same ethnic background, from different ethnic backgrounds, and from poorer countries outside Europe.

The results suggest that all the four classes share generalized attitudes towards immigrants ranging from being highly inclusive to being highly exclusive. In other words, even though one could expect finding classes of respondents with the combination of high, moderate, and low degrees of inclusive attitudes, the results revealed that respondents (in each class) weakly differentiated between the three groups of immigrants. Our results, therefore, may appear to be at odds with the fact that attitudes towards members of different outgroups spring from distinct cognitive, ideological, and motivational sources (see e.g., [94,95]). The reason behind the pattern of our results, however, might be that all the three types of immigrants may have already been perceived as low-status groups (as opposed to Western white expats for instance), that in turn elicited generalized attitudes towards them. The nature of that generalized attitude, whatever it is, may be what matters, that as discussed above, stems from the interplay between multiple factors including one’s basic personal values.

Community detection analysis revealed that Inclusive Europeans’ value structure seems to be the most *complex*, as opposed to Some, Few, and Exclusive Europeans, whose value structures are more simply divided into two large communities (social focus vs. personal focus). In the case of Inclusive class members however, the 10 basic values configured four separate communities, corresponding to the 4 higher-order value types proposed by Schwartz [35]: openness to change, conservation, self-transcendence, and self-enhancement. The only difference was that self-direction, instead of clustering with stimulation and hedonism, clustered with universalism and benevolence. As shown in Fig 1, self-direction value is adjacent to universalism, and after all, it is theoretically assumed to be motivationally close to universalism and benevolence (all belong to growth value types).

One explanation may be that Inclusive class members’ more complex value structure (in the sense of the number of communities) enables them to base their judgement on outgroup members through more egalitarian values (e.g., universalism and benevolence), that is less dependent on the causal effects of values tapping into opposing motivational goals (e.g., tradition, conformity). This highly resonates with the prevailing consensus in the literature that cognitive complexity is related both to lower degree of conservatism (see [96]) and more favorable intergroup attitudes [97,98]. Integrative complexity for example, captures the extent to which one both differentiates between opposing perspectives and integrates them into a coherent whole [99]. Inclusive network seems to be the most complex in this sense, as we see that 4 sets of values are differentiated from each other and integrated into four separate sets of values. Regarding the other three networks, however, in a less sophisticated manner, the two large communities consist of motivationally conflicting values. Future investigation is needed to directly test the relationship between the number and the nature of clusters in attitude networks and different forms of cognitive style including cognitive complexity.

Furthermore, the level of assortativity between the two categories of values (growth and self-protection) found for each class of respondents supplemented the results of the community detection analysis. As expected, the assortativity analysis showed that in the case of Inclusive class, values are more strongly interconnected within either growth value types or self-protection value types and the relationship between the two categories is comparatively weaker. In other words, regarding the most inclusive individuals, the values’ effects on each other are more confined within the limit of either more egalitarian or less egalitarian value types. This may imply that in terms of the inclusive individuals, values from dissimilar motivational goals, are less causally dependent upon each other, that in turn enables them to evaluate outgroup members on the basis of one’s universal concerns, more free from the restricting influence of ingroup interests. Moreover, the results showed that the global connectivity score (the overall level of interconnections), was found to be mostly invariant across the networks, implying that what makes the difference is the unique configuration of the 10 values for each class.

Prior research has documented that one’s intergroup attitudes are strongly reflected in the level of importance they place on holding certain value types (e.g., growth vs. self-protection). Theoretically speaking, the present study contributes to the literature in the field, by showing that people’s intergroup attitudes are also expressed in how their personal values tend to be interconnected. Moreover, among several prejudice reduction intervention strategies, cognitive and emotional training as well as moral and value education have been argued to be effective tools on combating intergroup prejudice (see [100,101]). Thus, one possible practical implication of the current study may be, for the future research, to investigate if promoting complex thinking in respect of one’s personal values would lead to more egalitarian intergroup attitudes. In other words, a prejudice reduction strategy may involve promoting conscious consideration of opposing perspectives with respect to one’s personal values and examining whether this in turn results in a more complex value structure and a more favorable attitude towards the outgroup members. Our study concludes that mapping the dynamic structure of basic human values or moral codes in relation to intergroup attitudes can provide informative conceptual frameworks as well as effective practical strategies to fight intergroup prejudice.

## Supporting information

S1 Fig Confidence intervals around the edge weights (Exclusive class). (TIF)

S2 Fig Confidence intervals around the edge weights (Few class). (TIF)

S3 Fig Confidence intervals around the edge weights (Some class). (TIF)

S4 Fig Confidence intervals around the edge weights (Inclusive class). (TIF)

S1 Table Conceptual definitions of the 10 basic values. (DOCX)

S2 Table Descriptive statistics and correlation between variables (Inclusive class). (DOCX)

S3 Table Descriptive statistics and correlation between variables (Some class). (DOCX)

S4 Table Descriptive statistics and correlation between variables (Few class). (DOCX)

S5 Table Descriptive statistics and correlation between variables (Exclusive class). (DOCX)

S6 Table Demographic information of the four classes. (DOCX)

S7 Table The proportion of class membership conditioned by country. (DOCX)
