# Supplementary figures and images for: Exploring inclusiveness towards immigrants as related to basic values: A network approach
Source: PLoS One. 2021 Dec 2;16(12):e0260624. doi: 10.1371/journal.pone.0260624 (PMC8638986; doi:10.1371/journal.pone.0260624)

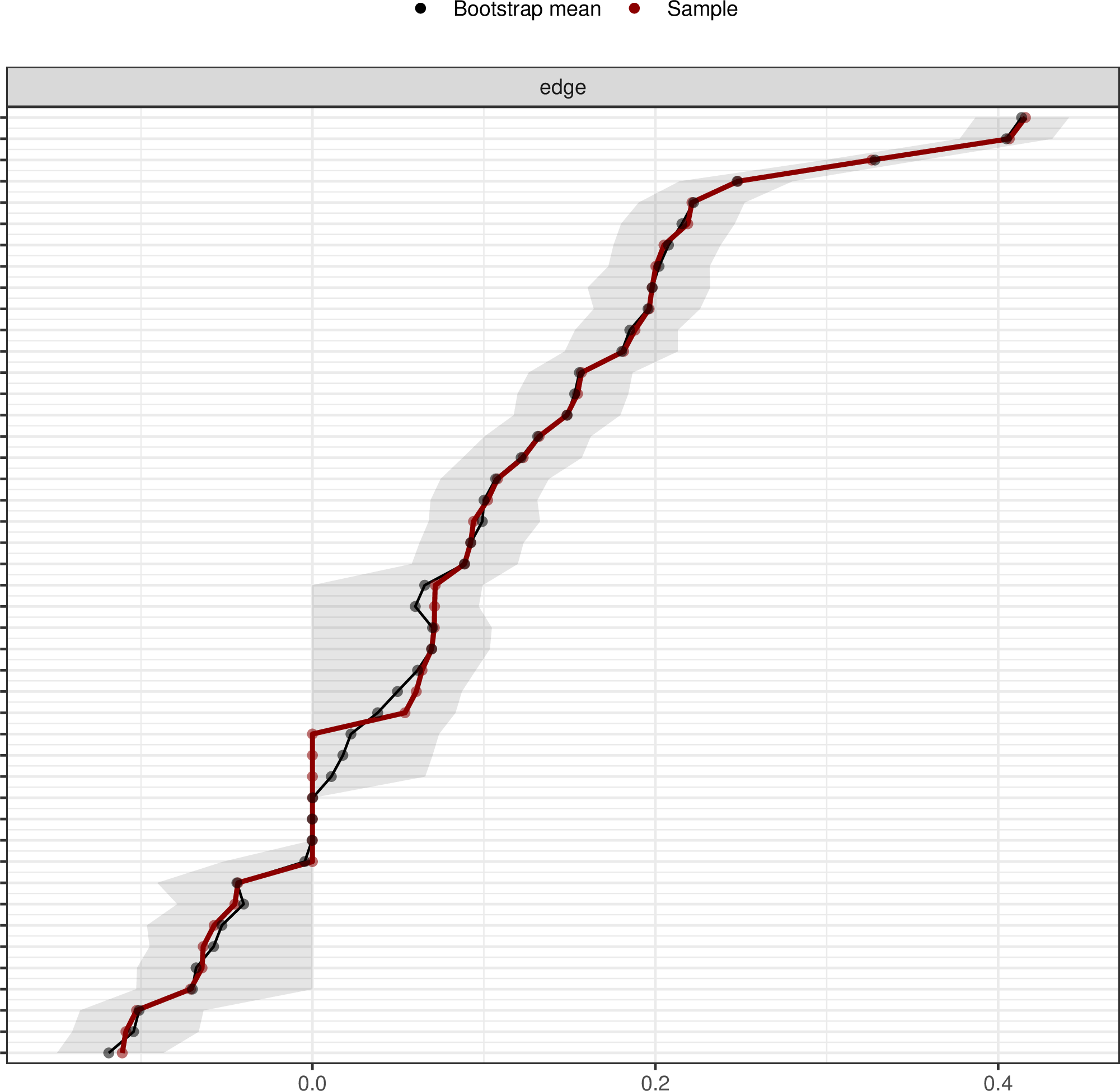

Supplement: S1 Fig — (TIF) [file pone.0260624.s001.tif]

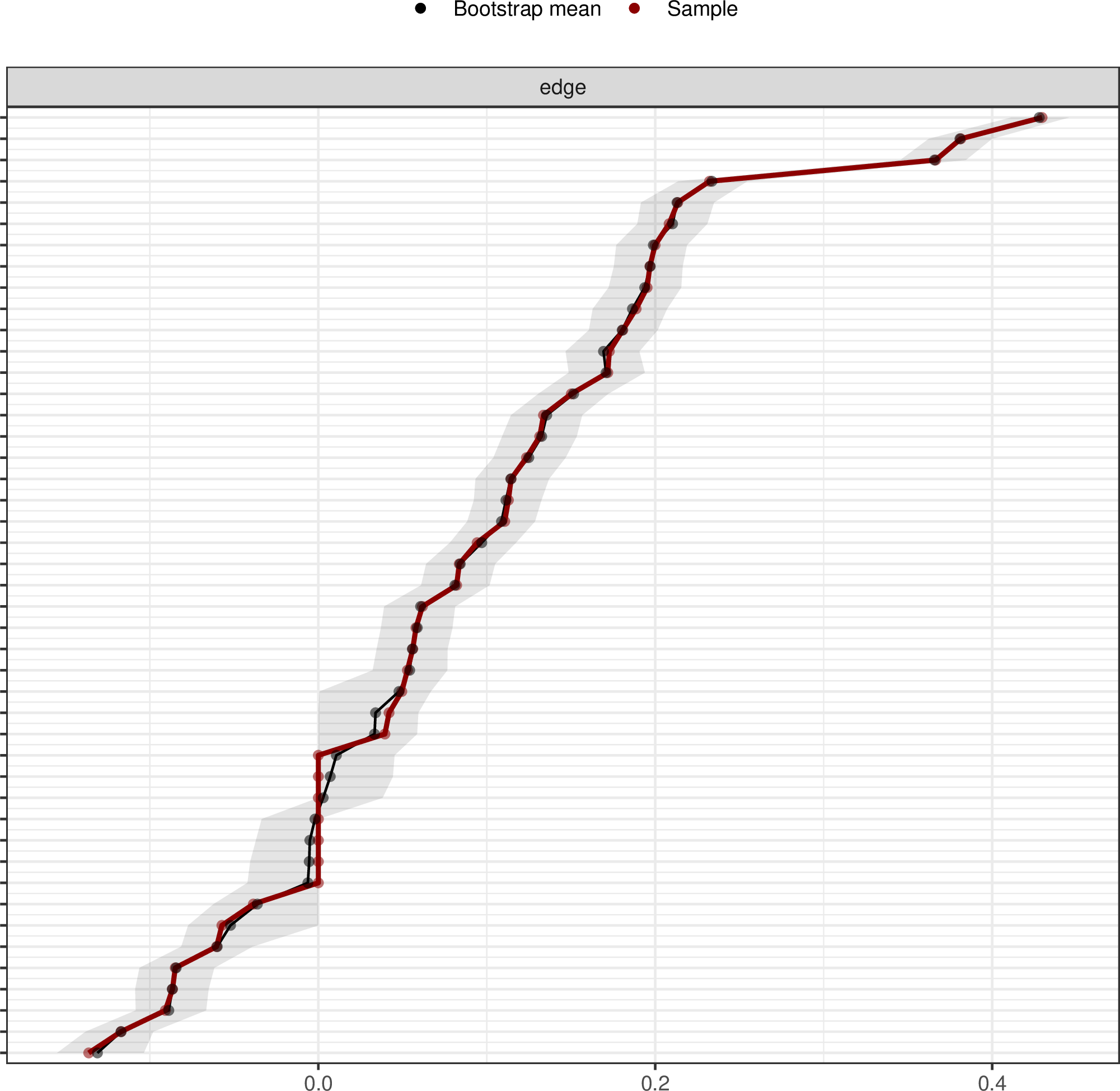

Supplement: S2 Fig — (TIF) [file pone.0260624.s002.tif]

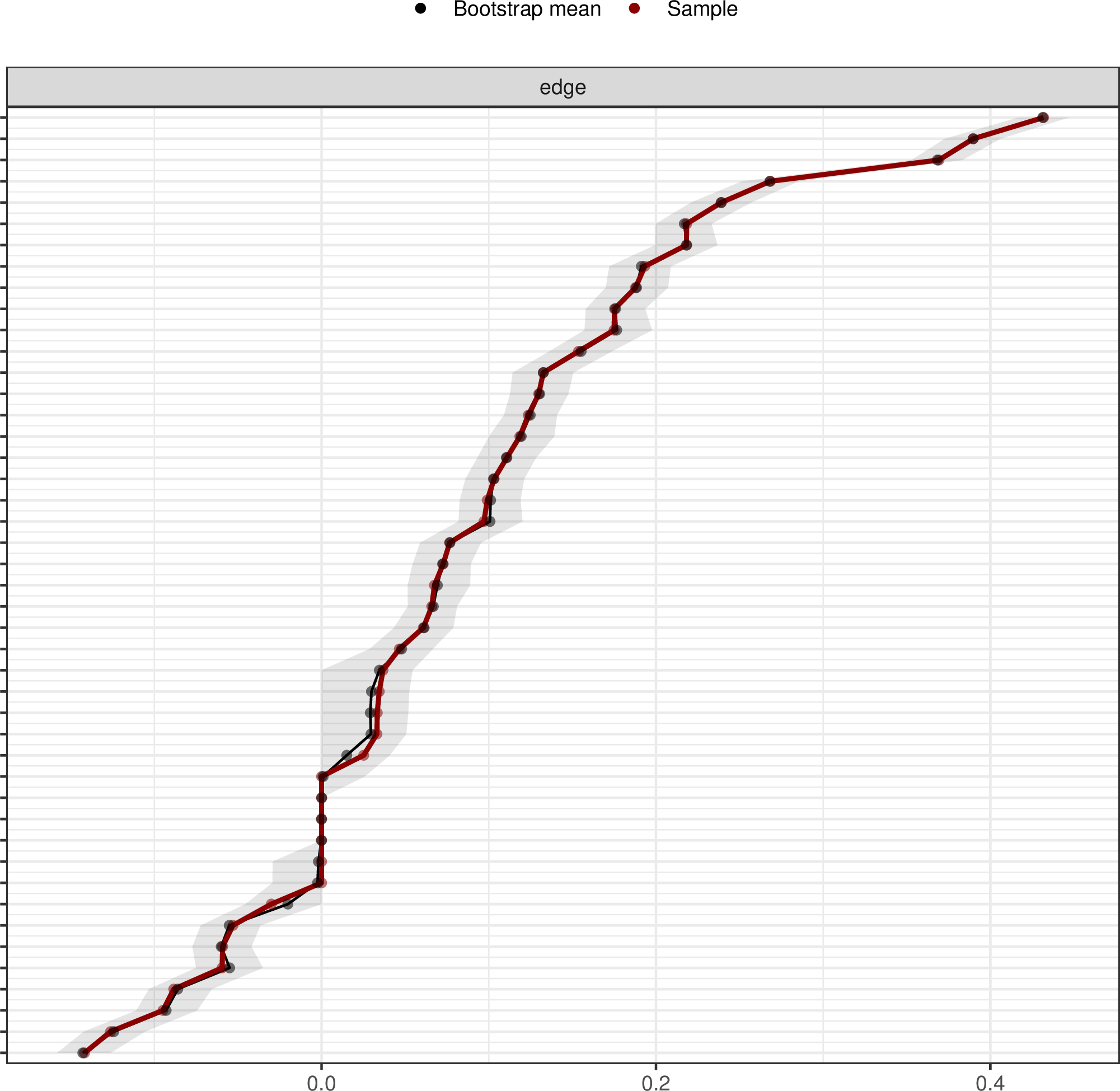

Supplement: S3 Fig — (TIF) [file pone.0260624.s003.tif]

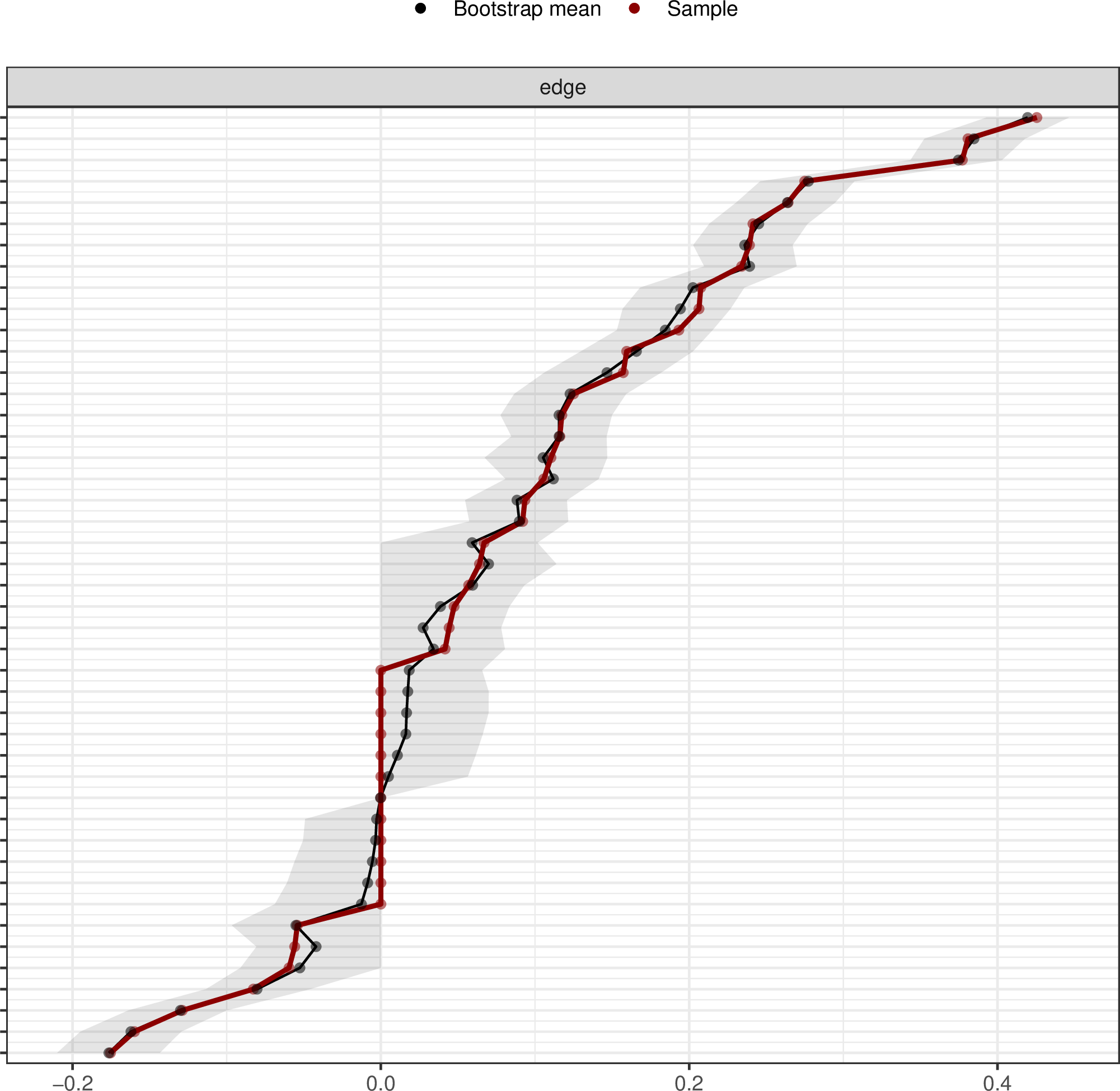

Supplement: S4 Fig — (TIF) [file pone.0260624.s004.tif]
