# Supplementary material for: Exploring inclusiveness towards immigrants as related to basic values: A network approach
Source: PLoS One. 2021 Dec 2;16(12):e0260624. doi: 10.1371/journal.pone.0260624 (PMC8638986; doi:10.1371/journal.pone.0260624)
Supplement: S1 Table — (DOCX) [file pone.0260624.s005.docx]

| Table S1. Conceptual definitions of the 10 basic values | |
| --- | --- |
| Value | Conceptual definition |
| Achievement | Personal success through demonstrating competence according to social standards. |
| Benevolence | Preservation and enhancement of the welfare of people with whom one is in frequent personal contact. |
| Conformity | Restraint of actions, inclinations, and impulses likely to upset or harm others and violate social expectations or norms. |
| Hedonism | Pleasure and sensuous gratification for oneself. |
| Power | Social status and prestige, control or dominance over people and resources. |
| Security | Safety, harmony, and stability of society, of relationships, and of self. |
| Self-Direction | Independent thought and action—choosing, creating, exploring. |
| Stimulation | Excitement, novelty, and challenge in life. |
| Tradition | Respect, commitment, and acceptance of the customs and ideas that traditional culture or religion provides. |
| Universalism | Understanding, appreciation, tolerance and protection for the welfare of all people and for nature. |
| *Note*. Source: Schwartz, S. H. (1994). Are there universal aspects in the structure and contents of human values?. *Journal of social issues*, 50, 19-45. | |
