# Supplementary material for: Exploring inclusiveness towards immigrants as related to basic values: A network approach
Source: PLoS One. 2021 Dec 2;16(12):e0260624. doi: 10.1371/journal.pone.0260624 (PMC8638986; doi:10.1371/journal.pone.0260624)
Supplement: S2 Table — (DOCX) [file pone.0260624.s006.docx]

| Table S2. Descriptive statistics and correlation between variables (Inclusive class) | | | | | | | | | | | | | | | | |
| --- | --- | --- | --- | --- | --- | --- | --- | --- | --- | --- | --- | --- | --- | --- | --- | --- |
| Variables | M | SD | 1. | 2. | 3. | 4. | 5. | 6. | 7. | 8. | 9. | 10. | 11. | 12. | 13. | 14. |
| 1. Political Interest | 2.38 | 0.97 |  |  |  |  |  |  |  |  |  |  |  |  |  |  |
| 2. Political Ideology | 4.11 | 2.19 | .10** |  |  |  |  |  |  |  |  |  |  |  |  |  |
| 3. imbgeco | 7.25 | 2.00 | -.22** | -.08** |  |  |  |  |  |  |  |  |  |  |  |  |
| 4. imueclt | 7.67 | 2.00 | -.20** | -.16** | .54** |  |  |  |  |  |  |  |  |  |  |  |
| 5. imwbcnt | 7.00 | 2.00 | -.17** | -.10** | .54** | .60** |  |  |  |  |  |  |  |  |  |  |
| 6. Security | 2.58 | 1.13 | -.20** | -.17** | .10** | .15** | .07** |  |  |  |  |  |  |  |  |  |
| 7. Conformity | 3.17 | 1.19 | -.16** | -.15** | .10** | .13** | .08** | .44** |  |  |  |  |  |  |  |  |
| 8. Tradition | 2.8 | 1.1 | -.16** | -.14** | .11** | .14** | .09** | .42** | .44** |  |  |  |  |  |  |  |
| 9. Benevolence | 1.82 | 0.71 | .06** | 0.02 | 0 | -.04** | -.06** | .25** | .17** | .27** |  |  |  |  |  |  |
| 10. Universalism | 1.85 | 0.69 | .17** | .12** | -.08** | -.16** | -.13** | .15** | .09** | .16** | .50** |  |  |  |  |  |
| 11. Self-direction | 2.25 | 0.96 | .18** | .04** | -.06** | -.07** | -.06** | .03* | -.12** | -.05** | .30** | .36** |  |  |  |  |
| 12. Stimulation | 3.31 | 1.22 | .03* | 0.03 | -.04** | -.06** | -.07** | -.05** | -.09** | -.08** | .20** | .19** | .43** |  |  |  |
| 13. Hedonism | 2.94 | 1.19 | 0.02 | .03* | 0.01 | -0.02 | 0 | .04** | -.06** | -.03* | .22** | .17** | .34** | .50** |  |  |
| 14. Achievement | 3.23 | 1.23 | -.04** | -.05** | .03* | .08** | 0.03 | .30** | .19** | .11** | .17** | .07** | .27** | .32** | .27** |  |
| 15. Power | 3.91 | 1.04 | -.09** | -.10** | 0 | .09** | .03* | .29** | .27** | .11** | .04** | -.07** | .16** | .21** | .24** | .53** |
| *Note*. ** = *p* < .01; * = *p* < .05. imbgeco = immigration is good or bad for economy; imueclt = whether immigration undermines or enriches culture; imwbcnt = immigration makes the country better or worse place to live. | | | | | | | | | | | | | | | | |
