# Supplementary material for: Exploring inclusiveness towards immigrants as related to basic values: A network approach
Source: PLoS One. 2021 Dec 2;16(12):e0260624. doi: 10.1371/journal.pone.0260624 (PMC8638986; doi:10.1371/journal.pone.0260624)
Supplement: S3 Table — (DOCX) [file pone.0260624.s007.docx]

| Table S3. Descriptive statistics and correlation between variables (Some class) | | | | | | | | | | | | | | | | |
| --- | --- | --- | --- | --- | --- | --- | --- | --- | --- | --- | --- | --- | --- | --- | --- | --- |
| Variables | M | SD | 1. | 2. | 3. | 4. | 5. | 6. | 7. | 8. | 9. | 10. | 11. | 12. | 13. | 14. |
| 1. Political Interest | 2.53 | .90 |  |  |  |  |  |  |  |  |  |  |  |  |  |  |
| 2. Political Ideology | 5.00 | 2.05 | 0 |  |  |  |  |  |  |  |  |  |  |  |  |  |
| 3. imbgeco | 5.83 | 2.02 | -.21** | -.02** |  |  |  |  |  |  |  |  |  |  |  |  |
| 4. imueclt | 6.04 | 2.15 | -.15** | -.08** | .55** |  |  |  |  |  |  |  |  |  |  |  |
| 5. imwbcnt | 5.56 | 1.89 | -.13** | -.05** | .56** | .63** |  |  |  |  |  |  |  |  |  |  |
| 6. Security | 2.35 | .97 | -.06** | -.03** | .06** | .01** | .07** |  |  |  |  |  |  |  |  |  |
| 7. Conformity | 2.97 | 1.06 | -.05** | -.06** | .04** | .07** | .05** | .41** |  |  |  |  |  |  |  |  |
| 8. Tradition | 2.69 | .98 | -.06** | -.06** | .09** | .10** | .07** | .38** | .41** |  |  |  |  |  |  |  |
| 9. Benevolence | 1.97 | .75 | .08** | .04** | -.01 | -.04** | -.03** | .31** | .20** | .34** |  |  |  |  |  |  |
| 10. Universalism | 2.12 | .72 | .13** | .11** | -.10** | -.13** | -.10** | .31** | .20** | .30** | .54** |  |  |  |  |  |
| 11. Self-direction | 2.40 | .92 | .13** | .01 | -.06** | -.05** | -.04** | .15** | 0 | .03** | .31** | .35** |  |  |  |  |
| 12. Stimulation | 3.47 | 1.16 | .02 | -.02** | -.02** | -.05** | -.06** | .01 | -.02** | -.04** | .17** | .17** | .42** |  |  |  |
| 13. Hedonism | 3.00 | 1.14 | .03** | 0 | -.02** | -.04** | -.03** | .10** | -.02* | .02* | .23** | .18** | .37** | .50** |  |  |
| 14. Achievement | 3.22 | 1.18 | -.01 | 0 | 0 | .03** | .02* | .27** | .18** | .07** | .15** | .12** | .30** | .36** | .30** |  |
| 15. Power | 3.77 | 1.00 | -.03** | -.07** | 0 | .05** | .04** | .22** | .23** | .06** | .04** | -.01 | .21** | .27** | .25** | .54** |
| *Note*. ** = *p* < .01; * = *p* < .05. imbgeco = immigration is good or bad for economy; imueclt = whether immigration undermines or enriches culture; imwbcnt = immigration makes the country better or worse place to live. | | | | | | | | | | | | | | | | |
