# Supplementary material for: Exploring inclusiveness towards immigrants as related to basic values: A network approach
Source: PLoS One. 2021 Dec 2;16(12):e0260624. doi: 10.1371/journal.pone.0260624 (PMC8638986; doi:10.1371/journal.pone.0260624)
Supplement: S4 Table — (DOCX) [file pone.0260624.s008.docx]

| Table S4. Descriptive statistics and correlation between variables (Few class) | | | | | | | | | | | | | | | | |
| --- | --- | --- | --- | --- | --- | --- | --- | --- | --- | --- | --- | --- | --- | --- | --- | --- |
| Variables | M | SD | 1. | 2. | 3. | 4. | 5. | 6. | 7. | 8. | 9. | 10. | 11. | 12. | 13. | 14. |
| 1. Political Interest | 2.78 | .87 |  |  |  |  |  |  |  |  |  |  |  |  |  |  |
| 2. Political Ideology | 5.45 | 2.15 | -.09** |  |  |  |  |  |  |  |  |  |  |  |  |  |
| 3. imbgeco | 4.35 | 2.16 | -.11** | .03** |  |  |  |  |  |  |  |  |  |  |  |  |
| 4. imueclt | 4.43 | 2.26 | -.07** | -.03** | .53** |  |  |  |  |  |  |  |  |  |  |  |
| 5. imwbcnt | 4.14 | 2.05 | -.05** | -.01 | .56** | .62** |  |  |  |  |  |  |  |  |  |  |
| 6. Security | 2.21 | .95 | .02* | 0 | .04** | .06** | .07** |  |  |  |  |  |  |  |  |  |
| 7. Conformity | 2.86 | 1.01 | .02* | -.02* | 0 | 0 | .01 | .38** |  |  |  |  |  |  |  |  |
| 8. Tradition | 2.61 | .97 | .03** | -.02 | .07** | .06** | .04** | .39** | .41** |  |  |  |  |  |  |  |
| 9. Benevolence | 2.12 | .82 | .09** | .02 | .03** | 0 | .03** | .43** | .29** | .42** |  |  |  |  |  |  |
| 10. Universalism | 2.31 | .78 | .09** | .05** | -.02 | -.06** | -.02 | .47** | .34** | .45** | .60** |  |  |  |  |  |
| 11. Self-direction | 2.54 | .97 | .14** | -.02* | -.01 | 0 | .01 | .25** | .07** | .10** | .37** | .37** |  |  |  |  |
| 12. Stimulation | 3.61 | 1.17 | .04** | -.03** | -.02* | -.03** | -.02* | .03** | -.01 | -.04** | .15** | .13** | .43** |  |  |  |
| 13. Hedonism | 3.12 | 1.19 | .07** | -.03** | -.02* | -.02* | 0 | .13** | 0 | 0 | .24** | .19** | .43** | .54** |  |  |
| 14. Achievement | 3.20 | 1.17 | .02* | -.02 | 0 | .03** | 0 | .25** | .16** | .08** | .21** | .18** | .36** | .41** | .34** |  |
| 15. Power | 3.65 | 1.02 | -.01 | -.04** | -.05** | .04** | 0 | .20** | .21** | .04** | .06** | .04** | .24** | .31** | .27** | .54** |
| *Note*. ** = *p* < .01; * = *p* < .05. imbgeco = immigration is good or bad for economy; imueclt = whether immigration undermines or enriches culture; imwbcnt = immigration makes the country better or worse place to live. | | | | | | | | | | | | | | | | |
