# Supplementary material for: Exploring inclusiveness towards immigrants as related to basic values: A network approach
Source: PLoS One. 2021 Dec 2;16(12):e0260624. doi: 10.1371/journal.pone.0260624 (PMC8638986; doi:10.1371/journal.pone.0260624)
Supplement: S5 Table — (DOCX) [file pone.0260624.s009.docx]

| Table S5. Descriptive statistics and correlation between variables (Exclusive class) | | | | | | | | | | | | | | | | |
| --- | --- | --- | --- | --- | --- | --- | --- | --- | --- | --- | --- | --- | --- | --- | --- | --- |
| Variables | M | SD | 1. | 2. | 3. | 4. | 5. | 6. | 7. | 8. | 9. | 10. | 11. | 12. | 13. | 14. |
| 1. Political Interest | 3.04 | .89 |  |  |  |  |  |  |  |  |  |  |  |  |  |  |
| 2. Political Ideology | 5.54 | 2.40 | -.10** |  |  |  |  |  |  |  |  |  |  |  |  |  |
| 3. imbgeco | 2.47 | 2.32 | -.08** | 0 |  |  |  |  |  |  |  |  |  |  |  |  |
| 4. imueclt | 2.54 | 2.29 | -.05** | -.02 | .64** |  |  |  |  |  |  |  |  |  |  |  |
| 5. imwbcnt | 2.50 | 2.27 | -.04** | 0 | .63** | .68** |  |  |  |  |  |  |  |  |  |  |
| 6. Security | 2.16 | .97 | .06** | .03* | .08** | .10** | .10** |  |  |  |  |  |  |  |  |  |
| 7. Conformity | 2.80 | 1.05 | .07** | .04* | .04** | .06** | .05** | .43** |  |  |  |  |  |  |  |  |
| 8. Tradition | 2.60 | 1.02 | .05** | .03* | .07** | .08** | .07** | .40** | .45** |  |  |  |  |  |  |  |
| 9. Benevolence | 2.29 | .92 | .10** | .03* | .08** | .07** | .08** | .46** | .39** | .45** |  |  |  |  |  |  |
| 10. Universalism | 2.51 | .85 | .06** | .07** | .08** | .04** | .05** | .50** | .43** | .47** | .60** |  |  |  |  |  |
| 11. Self-direction | 2.74 | 1.05 | .14** | -.03* | 0 | .01 | .01 | .24** | .13** | .08** | .34** | .34** |  |  |  |  |
| 12. Stimulation | 3.72 | 1.22 | .03** | -.03 | -.05** | -.05** | -.06** | 0 | .02 | -.06** | .14** | .13** | .43** |  |  |  |
| 13. Hedonism | 3.29 | 1.28 | .04** | -.04** | -.03* | -.05** | -.06** | .07** | 0 | -.03* | .21** | .18** | .45** | .56** |  |  |
| 14. Achievement | 3.23 | 1.22 | .07** | 0 | .03 | .03* | .01 | .19** | .23** | .09** | .24** | .23** | .38** | .43** | .32** |  |
| 15. Power | 3.57 | 1.09 | .01 | -.02 | -.05** | .01 | -.03* | .14** | .24** | .02 | .10** | .08** | .28** | .34** | .26** | .52** |
| *Note*. ** = *p* < .01; * = *p* < .05. imbgeco = immigration is good or bad for economy; imueclt = whether immigration undermines or enriches culture; imwbcnt = immigration makes the country better or worse place to live. | | | | | | | | | | | | | | | | |
