# Supplementary material for: Exploring inclusiveness towards immigrants as related to basic values: A network approach
Source: PLoS One. 2021 Dec 2;16(12):e0260624. doi: 10.1371/journal.pone.0260624 (PMC8638986; doi:10.1371/journal.pone.0260624)
Supplement: S6 Table — (DOCX) [file pone.0260624.s010.docx]

| Table S6. Demographic information of the four classes. | | | | |
| --- | --- | --- | --- | --- |
|  | Class | | | |
| Demographics | Inclusive | Some | Few | Exclusive |
| Gender (Women %) | 54.4 | 52.4 | 52.1 | 54.8 |
| Age (M) | 46.03 (17.51) | 50.66 (18.00) | 53.60 (18.07) | 55.23 (17.48) |
| Education years (M) | 14.56 (4.24) | 13.63 (4.02) | 12.43 (3.74) | 11.53 (3.51) |
| *Note*. The numbers in the parentheses represent standard deviations. | | | | |
|  |  |  |  |  |
