# Supplementary material for: Exploring inclusiveness towards immigrants as related to basic values: A network approach
Source: PLoS One. 2021 Dec 2;16(12):e0260624. doi: 10.1371/journal.pone.0260624 (PMC8638986; doi:10.1371/journal.pone.0260624)
Supplement: S7 Table — (DOCX) [file pone.0260624.s011.docx]

| Table S7. The proportion of class membership conditioned by country. | | | | |
| --- | --- | --- | --- | --- |
| Classes | Inclusive (%) | Some (%) | Few (%) | Exclusive (%) |
| Austria | 9.9 | 34.7 | 39.9 | 15.5 |
| Belgium | 15.8 | **57.6** | 20.9 | 5.6 |
| Bulgaria | 6.5 | 27.5 | 34.4 | 31.6 |
| Cyprus | 2.9 | 17.2 | **67.2** | 12.7 |
| Czechia | 1.4 | 22.5 | 39.7 | 36.4 |
| Estonia | 6.7 | 42.3 | 38.6 | 12.4 |
| Finland | 11.3 | 41.4 | 43.7 | 3.6 |
| France | 16.2 | 52.2 | 22.5 | 9.1 |
| Germany | 23.4 | 53.6 | 20.5 | 2.6 |
| Hungary | 1.8 | 13.8 | 44.3 | **40.1** |
| Ireland | 22.8 | 47.2 | 21.9 | 8.1 |
| Italy | 14.2 | 37.2 | 34.5 | 14.1 |
| Netherlands | 15.3 | 52.5 | 27.0 | 5.2 |
| Norway | **27.9** | 54.4 | 16.7 | 1.0 |
| Poland | 5.1 | 35.7 | 41.0 | 18.2 |
| Serbia | 20.1 | 31.3 | 26.9 | 21.7 |
| Slovenia | 9.3 | 51.4 | 29.8 | 9.5 |
| Switzerland | 17.3 | 56.2 | 23.4 | 3.1 |
| United Kingdom | 17.4 | 54.6 | 21.9 | 6.2 |
|  |  |  |  |  |
